# The impact of small intestinal bacterial overgrowth on the efficacy of fecal microbiota transplantation in patients with chronic constipation

**DOI:** 10.1128/mbio.02023-24

**Published:** 2024-08-28

**Authors:** Le Wang, Yue Xu, Long Li, Bo Yang, Di Zhao, Chen Ye, Zhiliang Lin, Jiaqu Cui, Yunkun Liu, Wanyong Zhu, Ning Li, Hongliang Tian, Qiyi Chen

**Affiliations:** 1 Department of Functional Intestinal Diseases, General Surgery of Shanghai Tenth People's Hospital, Tongji University School of Medicine, Shanghai, China; 2 Shanghai Gastrointestinal Microecology Research Center, Shanghai, China; 3 Shanghai Institution of Gut Microbiota Research and Engineering Development, Shanghai, China; College of Veterinary Medicine, Cornell University, Ithaca, New York, USA

**Keywords:** small intestinal bacterial overgrowth, fecal microbiota transplantation, chronic constipation, efficacy

## Abstract

To investigate the impact of Small Intestinal Bacterial Overgrowth (SIBO) on the efficacy of Fecal Microbiota Transplantation (FMT) in patients with chronic constipation, our research team included 218 patients with chronic constipation treated with FMT. Based on the results of the SIBO breath test, the patients were divided into two groups: the constipation with SIBO group (SIBO) and the constipation without SIBO group (non-SIBO). The efficacy of the two groups was evaluated using constipation-related scoring scales. At the same time, feces and small intestinal fluid samples were collected from both groups before and after FMT to compare the changes in the intestinal microbiota through 16S rRNA sequencing. In this study, it was found that the clinical efficacy of FMT in the SIBO group was superior to that in the non-SIBO group. After FMT treatment, both groups showed a significant increase in bowel frequency and improvement in stool characteristics. Abdominal symptoms, rectal symptoms, and defecation symptoms were significantly alleviated (*P* < 0.05), and patients’ quality of life was significantly enhanced (*P* < 0.05). After FMT, except for the Constipation Assessment Scale scores, other scale scores showed significant differences between the two groups, the SIBO group scoring significantly better than the non-SIBO group (*P* < 0.05). After FMT, there were minor changes in the colonic microbiota but more substantial changes in the small intestinal microbiota. At baseline, the SIBO group had a higher abundance of *Veillonella*, and lower abundances of *Escherichia-Shigella* and *Acinetobacter* compared to the non-SIBO group. Chronic constipation patients with SIBO have a better response to FMT than those without SIBO.

**IMPORTANCE:**

Existing studies have rarely considered the impact of the small intestine’s microbial state on the efficacy of fecal microbiota transplantation (FMT), nor have they extensively explored the effect of the small intestine’s microbial state on the recovery of colonic motility. Therefore, this study investigates the influence of small intestinal bacterial overgrowth (SIBO) on the efficacy of FMT in treating constipation, specifically the impact of the microbial state of the small intestine on the restoration of colonic homeostasis, and consequently on the recovery of colonic motility.

## INTRODUCTION

Small intestinal bacterial overgrowth (SIBO) is defined as the presence of an excessive number of bacteria in the small intestine that causes gastrointestinal symptoms. These bacteria are usually part of the colonic flora and mainly consist of Gram-negative aerobes and anaerobes that ferment carbohydrates to produce gas (1). The signs and symptoms of SIBO can be due to malabsorption of nutrients, alterations in intestinal permeability, inflammation, and immune activation caused by pathological bacterial fermentation in the small intestine (2). Symptoms primarily include nausea, bloating, abdominal pain, diarrhea, and constipation. In extreme cases, symptoms may include weight loss, anemia, deficiencies in fat-soluble vitamins, and inflammation of the small intestinal mucosa (3). The recent North American consensus accurately identified that a bacterial colony count of ≥10^3^ colony-forming units per milliliter in the aspirate from the duodenum/jejunum is the diagnostic criterion for SIBO (4). The breath test, which primarily measures hydrogen and methane gases, is currently the most commonly used method for diagnosing SIBO (5, 6).

Constipation is usually defined according to the Rome diagnostic criteria as a reduced bowel movement frequency (less than three times per week in adults), difficulty in evacuation (straining, a sensation of obstruction, or incomplete bowel movement), stool that is dry and hard, and the need for manual maneuvers to facilitate bowel movements. Constipation can be primary or secondary to other diseases, such as Parkinson’s disease (7). The global prevalence of chronic constipation in adults ranges from 8.2% to 32.9%, with the prevalence in the Chinese population being between 4.0% and 10.0%(8, 9). The microbiota diversity in patients with chronic constipation is different from that in healthy individuals. Research has found significant differences in the colonic mucosal microbiota structure between patients with chronic constipation and healthy individuals (10). There is a notable decrease in the relative abundance of two common probiotic genera, *Lactobacillus* and *Bifidobacteria*, in patients with chronic constipation. Additionally, the levels of two main microbial metabolites, short-chain fatty acids and secondary bile acids, are significantly lower in the feces of patients with chronic constipation compared to healthy individuals (11–13).

Fecal microbiota transplantation (FMT) is defined as the transfer of fecal material from a healthy donor into the intestinal tract of a recipient with minimal processing, aiming to restore the microbial community of the gut and treat diseases associated with alterations in the gut microbiome (14). The gut microbiota, mainly composed of bacteria, viruses, fungi, protozoa, and archaea, plays a crucial role in human health and disease. The disruption of the gut microbiota’s homeostasis can lead to dysbiosis, which is associated with a range of diseases such as constipation, inflammatory bowel disease (IBD), irritable bowel syndrome (IBS), colorectal cancer, metabolic syndrome, and autism (15, 16). Currently, FMT has become an established treatment for recurrent *Clostridium difficile* infection (rCDI) and is recommended by international guidelines (17). Studies have also found that FMT can provide a protective effect for patients at high risk of rCDI, with 78.1% of patients at risk of rCDI remaining free of CDI infection within 1 year post-transplant (18). In recent years, FMT has been increasingly applied in the treatment of digestive system diseases such as constipation, diarrhea, and IBS (19, 20). The efficacy of FMT varies greatly depending on the type of disease; for instance, FMT has shown better results in treating slow transit and mixed-type constipation compared to outlet obstruction-type constipation (21). The state of gut microbiota also influences the efficacy of FMT; however, there’s limited research on the impact of SIBO on the effectiveness of FMT in patients with chronic constipation. Thus, the purpose of this study is to explore the impact of SIBO on the efficacy of FMT in patients with chronic constipation.

## MATERIALS AND METHODS

### Patient and data collection

This study is part of research registered on ClinicalTrials.gov under the identifier NCT06208930, which included 218 patients with chronic constipation who underwent FMT in our department from November 2022 to December 2023. The study collected a total of 639 samples, including 389 fecal samples and 250 small intestine fluid samples. The basic information of the patients is shown in Table 1. All patients underwent breath testing for SIBO upon admission, and constipation-related questionnaires were administered before and after FMT [including the Bristol Stool Form Scale (BSFS), Constipation Assessment Scale (CAS), Constipation Scoring System (CSS), Patient Assessment of Constipation Symptoms (PAC-SYM), and Patient Assessment of Constipation Quality of Life (PAC-QOL)]. Fecal samples and small intestinal fluid from the patients were collected before each FMT and after FMT for 16S rRNA sequencing to assess the status of the gut microbiome.

**TABLE 1:** General information of the patients^*a*^^,^^*b*^

Characteristics	Overall (*N* = 218)
Age (mean ± standard deviation)	55.62 ± 16.48
Gender (male/female)	95/123
SIBO (positive/negative)	88/130
Disease duration (year)	13.46 ± 7.98
Complete Spontaneous Bowel Movements (CSBM) time/week	1.32 ± 0.66
Spontaneous Bowel Movements (SBM) time/week	1.76 ± 0.59
Wexner scale	16.27 ± 1.68
Bristol Stool Form Scale (BSFS)	1.30 ± 0.46
PAC-SYM	2.95 ± 1.32
PAC-QOL	2.89 ± 0.48
History of laxative use	
Irritant laxatives	94
Osmotic laxatives	70
Solvent laxatives	58
Lubricant laxatives	36
Taking probiotics	123
Enema	22

^*a*^ Inclusion criteria: (i) admitted with a diagnosis of chronic constipation; (ii) underwent SIBO breath testing upon admission; (iii) availability of fecal and small intestinal fluid samples before and after FMT.

^*b*^ Exclusion criteria: (i) presence of gastrointestinal malignancy; (ii) history of gastrointestinal surgery; (iii) presence of megacolon; (iv) history of extensive antibiotic use; (v) patients with chronic constipation due to other diseases.

### Observational indicators

#### Bowel movement frequency

Complete Spontaneous Bowel Movement (CSBM) refers to a bowel movement that the patient feels is satisfactory or relieving. Spontaneous Bowel Movement (SBM) is counted on a weekly basis to track the changes in CSBM and SBM before and after FMT.

### Stool form scale

The BSFS (22) is used to assign scores from 1 to 7 for stool types: type 1: separate hard lumps; type 2: sausage-shaped with lumps; type 3: sausage-shaped with cracks on the surface; type 4: smooth, soft sausage or snake; type 5: soft blobs with clear-cut edges; type 6: mushy consistency; type 7: watery, no solid pieces.

#### Constipation symptoms

##### Wexner scale

This scale includes eight items: bowel movement frequency, time per bowel movement, pain assessment, forms of assistance, completeness, number of unsuccessful attempts at bowel movement within 24 hours, abdominal pain, and duration of condition. Each is scored according to severity or frequency, ranging from 0 (none) to 4 (very severe), with higher scores indicating more severe constipation. A total score of 15 or above is considered indicative of constipation, with higher scores reflecting a more severe condition (23).

##### CAS

The CAS is a tool used to assess the severity of constipation symptoms. It is typically used in patients with chronic constipation, especially in the elderly or those in long-term care contexts. It covers eight items, including bowel movement frequency, stool consistency, difficulty of evacuation, and abdominal discomfort. Each is scored from 0 (no symptoms) to 2 (severe symptoms), with the sum of scores for all items producing a total score ranging from 0 to 16. A higher score indicates more severe constipation.

##### CSS

Proposed by Jorge and Wexner in 1993, the CSS is a scale for rating the severity of constipation. It involves scoring multiple symptoms in constipation patients to quantify the severity of constipation. Symptoms include frequency of bowel movements, straining, stool hardness, incomplete evacuation, obstructive sensation, manual maneuvers (such as the use of fingers), use of laxatives, and number of bowel movements per week. A higher total score indicates more severe constipation.

##### PAC-SYM

This self-assessment scale for constipation symptoms includes three dimensions—abdominal, rectal, and stool symptoms—with 12 items in total. It uses a 5-point Likert scale ranging from 0 (none) to 4 (very severe), with each dimension’s score being the average of the scores for all symptoms in that dimension, and the total score being the average of all 12 items. Higher scores represent more severe constipation symptoms.

### Quality of life assessment

Quality of life is assessed using the PAC-QOL questionnaire (24) before and after treatment. The questionnaire includes 28 items, divided into four dimensions: physical discomfort, psychosocial discomfort, worries and concerns, and satisfaction. Each item is rated on a 5-point scale from 0 (not at all) to 4 (extremely). The score for each dimension is the average of all items within that dimension, and the overall score is the average of all 28 items. Higher scores indicate a lower quality of life.

### Results and definitions

Record adverse events (AEs) during hospitalization for FMT. AEs refer to any new symptoms that arise during the FMT process, worsening of previous symptoms, and abnormal laboratory test results, which are classified into mild, moderate, and severe based on patient symptoms. Clinical cure is defined as autonomous defecation frequency of ≥3 times per week, a BSFS score of 3–5, and the absence of abdominal symptoms; clinical improvement is defined as an increased autonomous defecation frequency compared to admission, but less than three times per week, or reduced abdominal symptoms; clinical failure is defined as no increase in autonomous defecation frequency compared to admission, with persistent abdominal symptoms.

### Breath test

Before undergoing the breath test, patients must fast for 12 hours. The test method refers to the consensus method (4, 6). If the patient’s test results show any one or more of the following conditions: CH_4_ (methane) ≥10 ppb, H_2_ (hydrogen) ≥20 ppb, or NO (nitric oxide) ≥10 ppb, then the breath test result is considered positive. Otherwise, it is negative.

### The donor screening

Donor selection should meet the following criteria: (i) age between 18 and 30 years; (ii) body mass index of 18–25 kg/m^2^; (iii) no pathological signs during physical examination; (iv) no history of infectious diseases; (v) no recent gastrointestinal, metabolic, neurological history, or other systemic diseases; (vi) no recent use of drugs that could damage the composition of the gut microbiota; (vii) regular healthy diet, appropriate exercise, harmonious family environment, and no smoking or drinking habits; and (viii) passing blood and stool tests before donating feces, including general blood and stool tests as well as potential pathogen or infectious disease screenings.

### Preparation and procedure of FMT

Approximately 100 g of donated feces is collected into a sterile container, to which 300 mL of saline is added. The mixture is then stirred to allow it to pass through 2.0 mm and 0.5 mm mesh filters. Sterile glycerol is added to reach a final concentration of 10%, and the solution is stored at −20°C for 1–8 weeks until use. Patients receive a polyethylene glycol bowel lavage 12–24 hours before FMT. The fecal suspension is thawed in a 37°C water bath and is infused into the patient through a nasoenteric tube placed in advance, within 6 hours after thawing.

### DNA extraction and 16S rRNA gene sequencing

Microbial genomic DNA from fecal samples was extracted using the PowerMax Extraction Kit (MoBio Laboratories, Carlsbad, CA, USA), following the manufacturer’s protocol. Subsequently, the quantity and purity of the microbial DNA were assessed using agarose gel electrophoresis and a NanoDrop ND-1000 spectrophotometer (Thermo Fisher Scientific, Waltham, MA, USA). To amplify the V4 regions of 16S rRNA, we used two universal primers, specifically 515 forward primer (5′-GTGYCAGCMGCCGCGGTAA-3′) and 806 reversed primer (5′-GGACTACNVGGGTWTCTAAT-3′). The polymerase chain reaction (PCR) was performed within a 50 µL reaction mixture. The PCR cycle consisted of an initial denaturation at 98°C for 30 seconds, followed by 25 cycles that included denaturation at 98°C for 15 seconds, annealing at 58°C for 15 seconds, extension at 72°C for 15 seconds, and concluded with a final extension at 72°C for 1 minute. Subsequently, the PCR products were purified using AMPure XP Beads (Beckman Coulter, Indianapolis, IN), and the DNA concentration was measured with the PicoGreen double-stranded DNA Assay Kit (Invitrogen, Carlsbad, CA, USA). Following the quantification, the DNA libraries were sequenced on an Illumina NovaSeq 6000 platform with a 2 × 250 bp pair-end configuration; this sequence was performed at Shanghai Biotecan Pharmaceuticals Co., Ltd. (Shanghai, China). The amplicons were purified using an AxyPrep DNA Gel Extraction Kit (Axygen Biosciences, Union City, CA) followed by library quantification using a Qubit dsDNA BR Assay Kit (Thermo Fisher Scientific). Finally, the pooled amplicons were paired-end sequenced (2 × 250 bp) on an Illumina HiSeq PE250 sequencing platform. The sequencing depth was 42,501 reads per sample.

### Data processing, analysis, and visualization

We utilized Qiime2 version 2023.2.0 to conduct DADA2 processing on the raw sequence data (25, 26). First, we executed quality filtering to eliminate adapter and barcode sequences, and trimmed sequences to an appropriate length to discard sequences with an average quality score below 25. The sequences were then dereplicated and assessed for sequence variants, merged, and, lastly, evaluated for chimeric sequences following the standard DADA2 method. We excluded any amplicon sequence variant (ASV) that had a frequency of less than 50 in all samples or that appeared in less than three samples. Post filtration, representative sequences and biom-formatted tables were labeled using the Greengenes2 2022.10 database C. The resulting table and taxonomy artifacts were exported as a biom table and text file, respectively, for additional analyses subsequent to the integration of taxa data into the biom-formatted ASV table.

We calculated and visualized the alpha-diversity and beta-diversity indices using the “microeco” package (v.0.15.0) (27). The principal coordinate analysis (PCoA), taxonomic composition bar plot, feature abundance box plot, Venn plot, and heatmap plot were also generated using this package. Given that the microbiota was expressed as relative abundance, we utilized linear discriminant analysis (LDA) effect size (LEfSe) analysis to compare differences in microbiota composition (28). We applied the Phylogenetic Investigation of Communities by Reconstruction of Unobserved States (v.2.5.1) workflow to predict the metagenome functions of the microbiota, and the functional pathways were annotated using the Kyoto Encyclopedia of Genes and Genomes (KEGG) database (29).

### Statistical analysis

In this study, continuous variables between two groups were analyzed using the *t*-test, while continuous variables among multiple groups were assessed using analysis of variance (also known as the F-test). All categorical and ordinal variables were evaluated using the χ^2^ test, with the results expressed in frequencies and percentages. The statistical tool used for analysis was SPSS version 26.0.

## RESULTS

### The safety of FMT for constipation patients with SIBO

During the treatment and follow-up period of FMT, 11.36% (10/88) of patients in the SIBO group experienced 15 AEs (see Table 2), with bloating being the most common, occurring in 5 cases, and the rest including 4 cases of abdominal pain, 2 cases of diarrhea, 2 cases of nausea and vomiting, and 2 cases of fever; 13.08% (17/130) of patients in the non-SIBO group experienced 24 AEs, with bloating being the most common, occurring in 10 cases, and the rest including 8 cases of abdominal pain, 1 case of diarrhea, 2 cases of nausea and vomiting, and 3 cases of fever. These AEs disappeared or were alleviated after symptomatic treatment, and there were no FMT-related deaths.

**TABLE 2:** Two groups of adverse events

Adverse events	Non-SIBO n (n/N%) (*N* = 130)	SIBO n (n/N%) (*N* = 88)
Frequency		
Any adverse event	24 (100)	15 (100)
Any serious adverse event	0 (0)	0 (0)
Severity		
Any mild	19 (79.17)	11 (73.33)
Any moderate	5 (20.83)	4 (26.67)
Any severe	0 (0)	0 (0)
Any life threatening	0 (0)	0 (0)
Any death	0 (0)	0 (0)
Types of adverse events		
Abdominal bloating	10 (41.67)	5 (33.33)
Abdominal pain	8 (33.33)	4 (26.67)
Diarrhea	1 (4.17)	2 (13.33)
Nausea and vomiting	2 (8.33)	2 (13.33)
Fever	3 (12.5)	2 (13.33)

### Constipation patients with SIBO experience better efficacy from FMT than those without SIBO

In this study, it was found that the clinical efficacy in the SIBO group was superior to that in the non-SIBO group. Specifically, the cure rate was significantly higher in the SIBO group (*P*=0.003), and the rate of non-response was significantly lower compared to the non-SIBO group, with statistical significance (*P*=0.0001). However, there was no significant difference in the rate of symptom relief between the two groups (*P*=0.38) (see Table 3). After FMT treatment, there was a significant increase in the frequency of defecation in both groups, with an increase in the number of CSBM and SBM per week (*P* < 0.05) (Table 4). Furthermore, the stool characteristics improved noticeably, as shown by the increased BSFS scores (*P* < 0.05). Abdominal symptoms, rectal symptoms, and defecation symptoms also improved significantly. These improvements were assessed using various scales, including the PAC-SYM, the Wexner score, the CAS, and the CSS, with each showing statistically significant changes (*P* < 0.05) (Tables 5 and 6). The patients’ quality of life improved considerably, as indicated by significant changes in the scores on the PAC-QOL questionnaire (*P* < 0.05) (Table 7). Upon intergroup difference analysis, all scoring scales showed significant differences after FMT between the two groups, except for the CAS, with the SIBO group scoring better than the non-SIBO group (*P* < 0.05)

**TABLE 3:** Overall clinical efficacy of both groups

	Non-SIBO % (n/N)	SIBO % (n/N)	*P*
Cure rate	35.38 (46/130)	55.68 (49/88)	0.003
Remission rate	20.00 (26/130)	25.00 (22/88)	0.38
Inefficiency	44.62 (58/130)	19.32 (17/88)	0.0001
Clinical response rate	55.38 (72/130)	80.68 (71/88)	0.0001

**TABLE 4:** Frequency of defecation before and after FMT in both groups

	Non-SIBO		SIBO		F	*P*
	Pre-FMT	Post-FMT	Pre-FMT	Post-FMT		
SBM time/week	1.88 ± 0.60	3.13 ± 1.58	1.67 ± 0.61	3.84 ± 1.60^a^	54.03	0.001
CSBM time/week	1.41 ± 0.66	2.40 ± 1.12	1.27 ± 0.64	2.94 ± 1.29^a^	51.34	0.001

^a^ Indicates a significant difference in scale scores after FMT in the SIBO group compared to the non-SIBO group, *P* < 0.05.

**TABLE 5:** PAC-SYM scores before and after treatment in both groups

	Non-SIBO		SIBO		F	*P*
	Pre-FMT	Post-FMT	Pre-FMT	Post-FMT		
Abdominal symptom	3.01 ± 0.78	1.71 ± 1.12	2.91 ± 0.71	1.23 ± 1.06^a^	54.189	0.001
Rectal symptom	2.98 ± 0.69	1.76 ± 1.10	2.84 ± 0.69	1.18 ± 1.00^a^	61.68	0.047
Defecation symptom	3.02 ± 0.69	1.66 ± 1.11	3.07 ± 0.66	1.26 ± 1.04^a^	62.51	0.001
Total scores	3.00 ± 0.38	1.71 ± 1.00	2.94 ± 0.43	1.22 ± 0.95^a^	86.83	0.002

^a^ Indicates a significant difference in scale scores after FMT in the SIBO group compared to the non-SIBO group, *P* < 0.05.

**TABLE 6:** Scores of clinical score scale before and after treatment in two groups

	Non-SIBO		SIBO		F	*P*
	Pre-FMT	Post-FMT	Pre-FMT	Post-FMT		
BSFS	1.30 ± 0.74	2.52 ± 0.83	1.32 ± 0.47	2.94 ± 0.92^a^	102.01	0.001
Wexner	16.37 ± 1.85	11.03 ± 3.92	16.57 ± 1.49	9.15 ± 3.90^a^	118.17	0.001
CAS	10.06 ± 1.79	5.11 ± 2.77	10.27 ± 1.83	4.49 ± 2.53	145.45	0.003
CSS	20.30 ± 3.78	10.96 ± 5.59	21.12 ± 3.32	8.59 ± 5.67^a^	140.56	0.007

^a^ Indicates a significant difference in scale scores after FMT in the SIBO group compared to the non-SIBO group, *P* < 0.05.

**TABLE 7:** PAC-QOL scores of quality of life before and after treatment in both groups

	Non-SIBO		SIBO		F	*P*
	Pre-FMT	Post-FMT	Pre-FMT	Post-FMT		
Physical discomfort	3.04 ± 0.69	1.76 ± 1.05	3.12 ± 0.66	1.44 ± 0.98^*a*^	55.401	0.001
Psychosocial discomfort	2.97 ± 0.58	1.66 ± 1.02	2.96 ± 0.59	1.34 ± 0.89^*a*^	63.68	0.002
Worry and anxiety	2.91 ± 0.52	1.98 ± 1.13	2.99 ± 0.56	1.41 ± 1.03^*a*^	52.062	0.016
Satisfaction	3.02 ± 0.57	1.58 ± 0.94	2.87 ± 0.57	1.09 ± 0.87^*a*^	123.935	0.027
Total scores	2.99 ± 0.31	1.74 ± 0.92	2.98 ± 0.27	1.32 ± 0.84^*a*^	132.722	0.001

^*a*^ Indicates a significant difference in scale scores after FMT in the SIBO group compared to the non-SIBO group, *P* < 0.05.

### Changes in gut microbiota after FMT in two groups of patients: the SIBO group and the non-SIBO group

**Fig 1:**
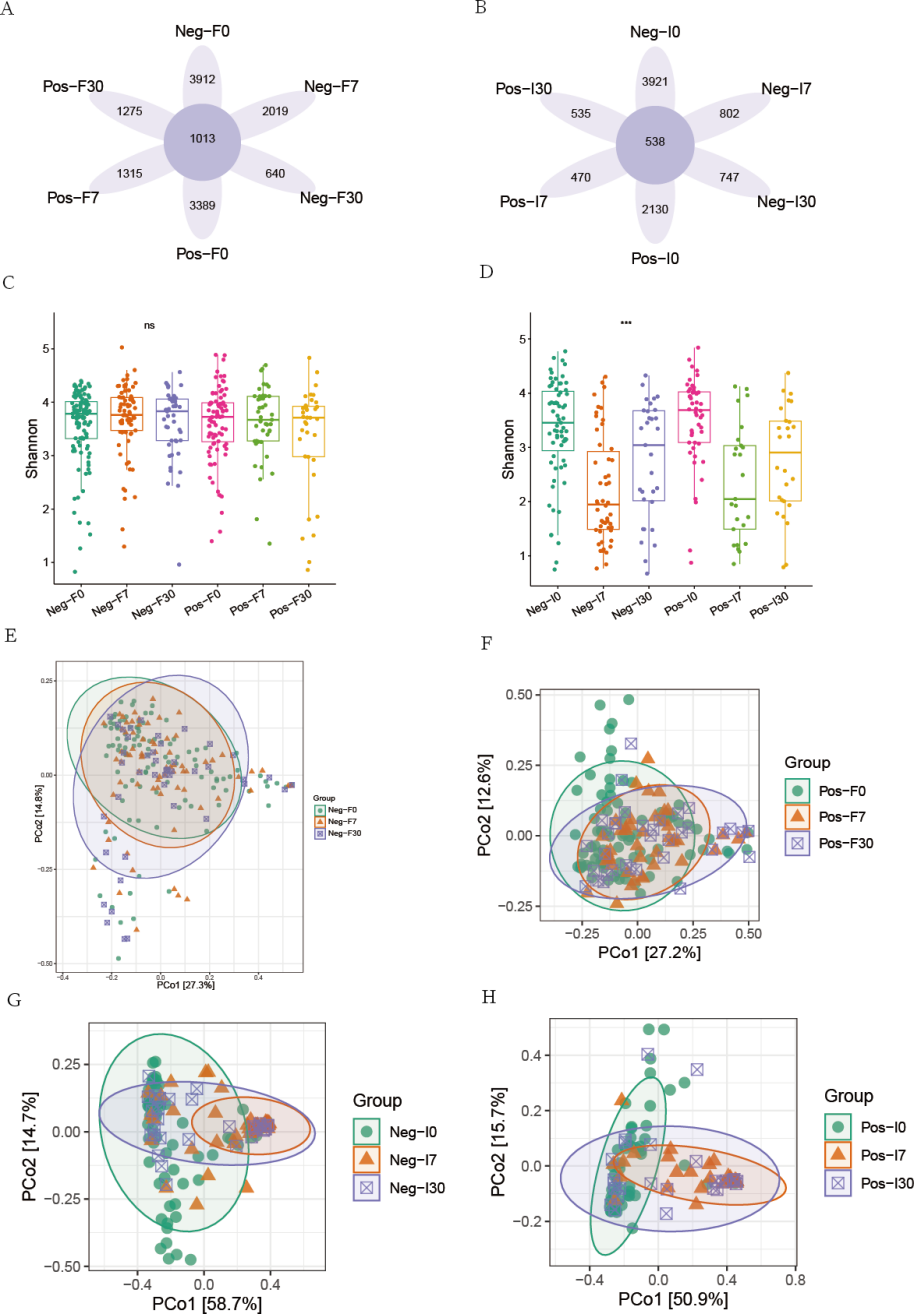
Overview of the microbiota structure. Venn plot illustrating the unique and shared ASVs of colonic (A) and small intestinal microbiota (B). Comparisons of Shannon index of colonic (C) and small intestinal microbiota (D). Beta-diversity of colonic (E and F) and small intestinal microbiota (G and H) between these groups using weighted Unifrac PCoA. “Neg” represents the non-SIBO group, “Pos” represents the SIBO group. “F” represents feces, “I” represents small intestinal fluid. “0” represents pre-FMT, “7” represents 7 days after FMT, and “30” represents 30 days after FMT.

The 16S rRNA gene sequencing data of fecal and small intestine fluid samples from two groups of patients were compared and analyzed to illustrate the differences before and after FMT. According to the Venn plot (Fig. 1A), in the fecal samples, the non-SIBO group had 3912, 2,019, and 640 unique ASVs before FMT, 7 days and 30 days after FMT, respectively, while the SIBO group had 3,389, 1,315, and 1,275 unique ASVs at the corresponding time points, while 1,013 ASVs were found to be present in all groups. In the small intestine fluid, there were 538 ASVs common to both the SIBO and non-SIBO groups before and after FMT(Fig. 1B). The alpha- and beta-diversity analyses were performed to illustrate the microbial community richness and composition before and after FMT in both groups of patients. There was no significant difference in the alpha-diversity of microbes in the feces of both groups before and after FMT (Fig. 1C), while the alpha-diversity of microbes in the small intestine fluid showed a decreasing trend after FMT (Fig. 1D). The beta-diversity analysis using the weighted UniFrac distance (Fig. 1E through H) showed that the beta-diversity of feces changed a little before and after FMT, especially in the non-SIBO group (Fig. 1E and F); the beta-diversity of small intestine fluid changed more significantly before and after FMT, particularly in the SIBO group (Fig. 1G and H).

**Fig 2:**
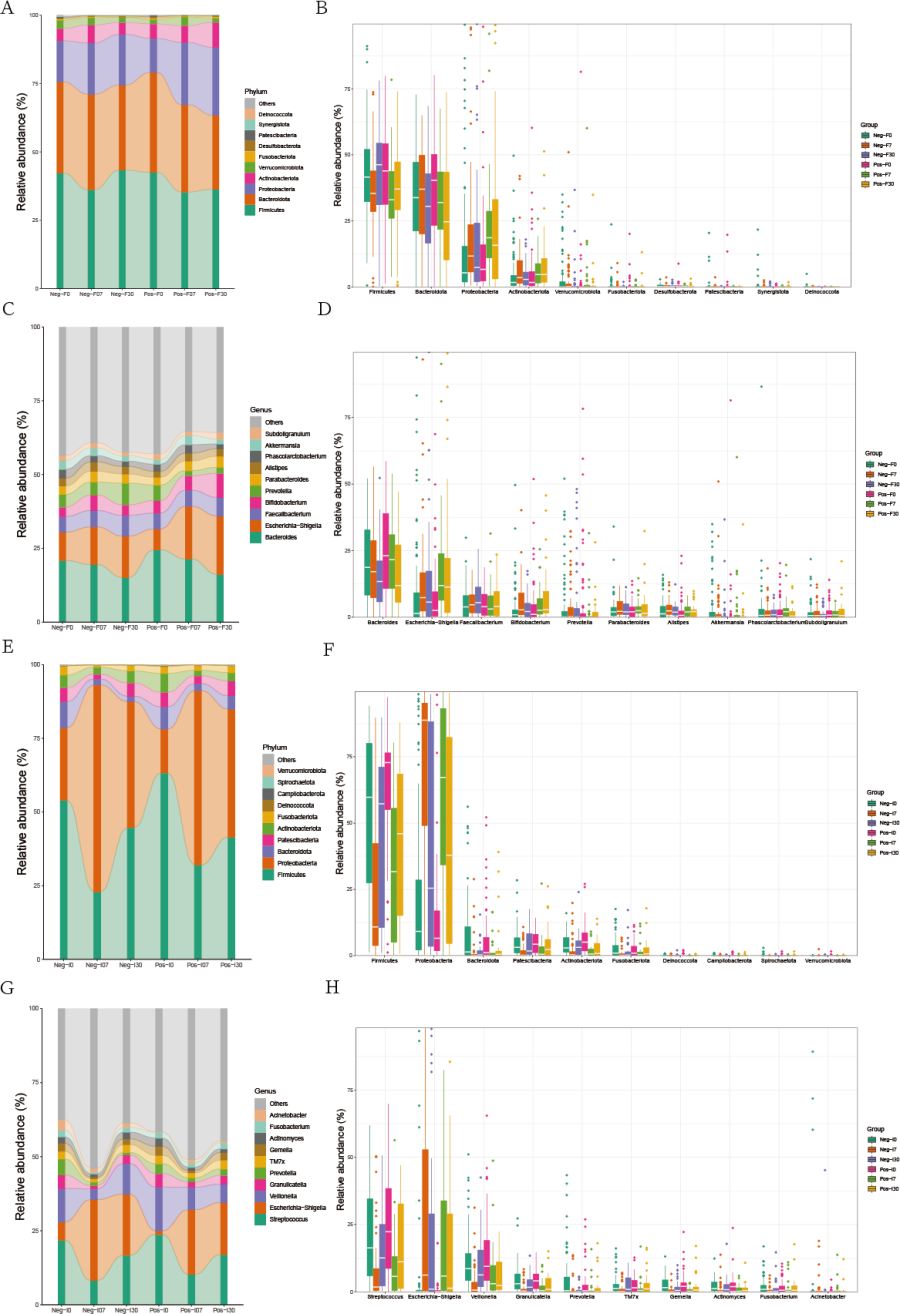
Bar plot and box plot of the top 10 most abundant species at different levels in the colon and small intestinal microbiota. (**A** and **B**) Phylum level in the colon microbiota. (**C** and **D**) Genus level in the colon microbiota. (**E** and **F**) Phylum level in the small intestinal microbiota. (**G** and **H**) Genus level in the small intestinal microbiota. “Neg” represents the non-SIBO group, “Pos” represents the SIBO group. “F” represents feces, “I” represents small intestinal fluid. “0” represents pre-FMT, “7” represents 7 days after FMT, and “30” represents 30 days after FMT.

Figure 2 presents the changes in the composition of the colonic and small intestinal microbiota of the two groups at different taxonomic levels before and after FMT. We found that the top 4 abundant at the phylum in the colonic microbiota were *Firmicutes*, *Bacteroidota*, *Proteobacteria*, and *Actinobacteriota*, but the changes before and after FMT were minor in both groups (Fig. 2A and B). At the genus level, the top 4 abundant microbiota were *Bacteroides*, *Escherichia-Shigella*, *Faecalibacterium*, and *Bifidobacterium*. In the non-SIBO group, the abundance of these microbiota did not change significantly before and after FMT, but in the SIBO group, the abundance of *Escherichia-Shigella* and *Bifidobacterium* showed a significant upward trend, especially *Bifidobacterium*, whose abundance increased from 4.29% to 4.94% 7 days after FMT and further increased to 8.21% 30 days after FMT (Fig. 2C and D). The small intestinal microbiota underwent more significant changes. At the phylum level, the top 4 abundant phylum in the small intestinal microbiota were *Firmicutes*, *Proteobacteria*, *Bacteroidota*, and *Patescibacteria*. In both groups, *Firmicutes* showed a significant downward trend 7 days after FMT, but in the non-SIBO group, there was a noticeable upward trend 30 days after FMT, while the upward trend was less pronounced in the SIBO group. Compared to before FMT, the abundance of *Firmicutes* in the SIBO group decreased from 6.30% to 4.14%, and in the non-SIBO group, it decreased from 5.36% to 4.47%. The trend of *Proteobacteria* in both groups was opposite to that of *Firmicutes* (Fig. 2E and F). At the genus level, the abundance trend of *Streptococcus* was similar in both groups, showing a significant downward trend 7 days after FMT, but an upward trend 30 days after FMT, whereas the abundance of *Escherichia-Shigella* markedly increased after FMT in both groups (Fig. 2G and H).

**Fig 3:**
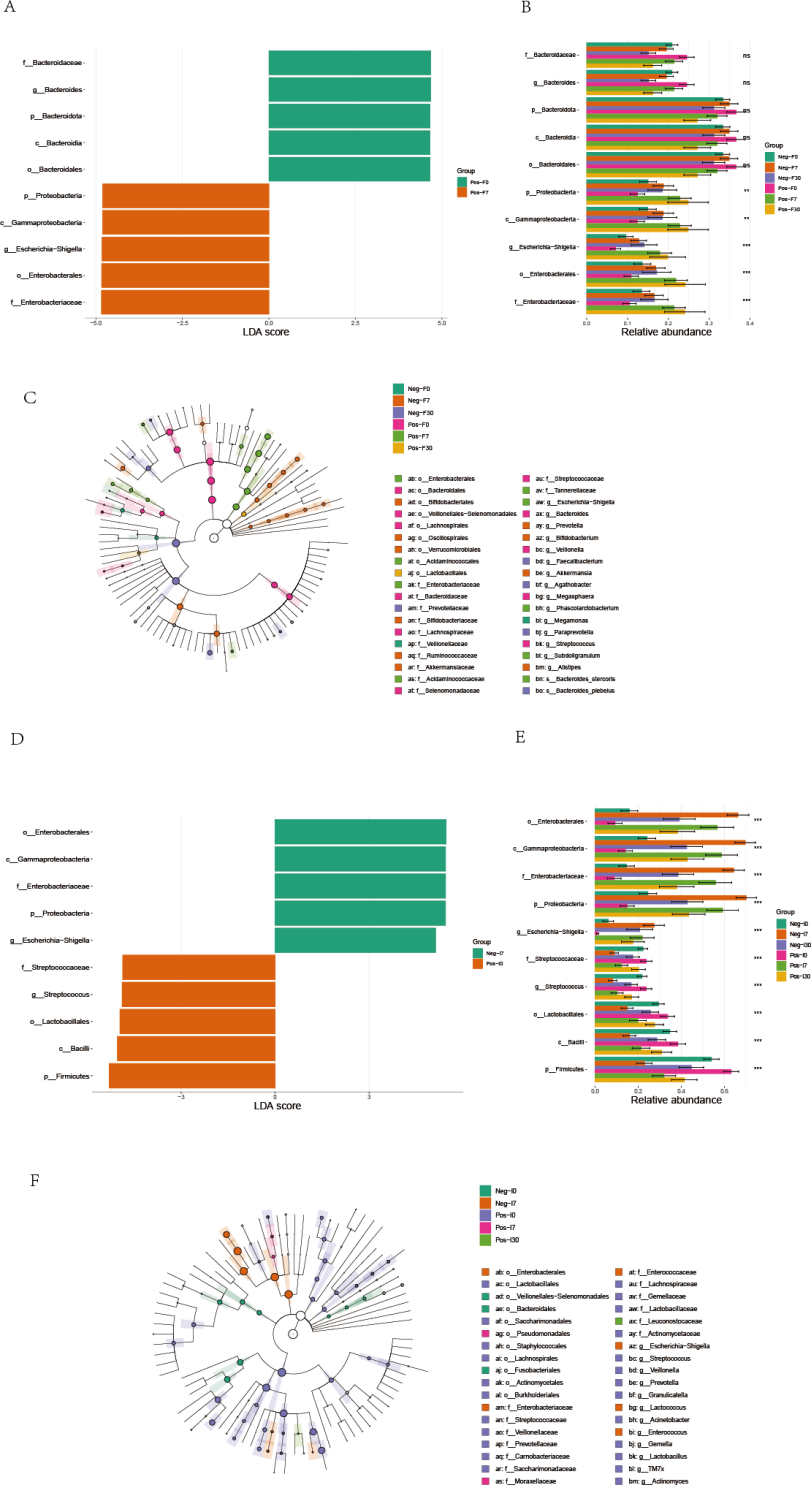
Represents the LEfSe analysis of the colon microbiota and small intestinal microbiota for two groups of patients. The LEfSe analysis was performed using an LDA score threshold of >2.0 to determine the differences in the colon microbiota (panels A and B) and their cladogram (panel C) before and after FMT in the two groups. It also examines the differences in the small intestinal microbiota (panels D and E) and their cladogram (panel F) between the two groups. “Neg” represents the non-SIBO group, “Pos” represents the SIBO group. “F” denotes feces, “I” denotes small intestinal fluid. “0” represents pre-FMT, “7” represents 7 days after FMT, and “30” represents 30 days after FMT.

Next, we aimed to identify the differences in specific taxonomic groups in the feces and small intestinal fluid before and after transplantation for both groups. Therefore, we conducted a LEfSe analysis, which utilizes the magnitude of effect size to enrich the bacterial clades that vary in abundance between the two groups. Under the significance threshold (*P* < 0.05) and an LDA score >2, Fig. 3A through D, respectively, display the taxonomic units of the colonic and small intestinal microbiota that differ in abundance. Additionally, we generated cladograms from the LEfSe analysis to provide a visual result of the phylogenetic distribution of these samples from the class to the genus level, with the size of each circle in the cladogram representing the abundance of certain taxonomic groups (Fig. 3B and D).

### The baseline state of the small intestinal microbiota affects the efficacy of FMT treatment

**Fig 4:**
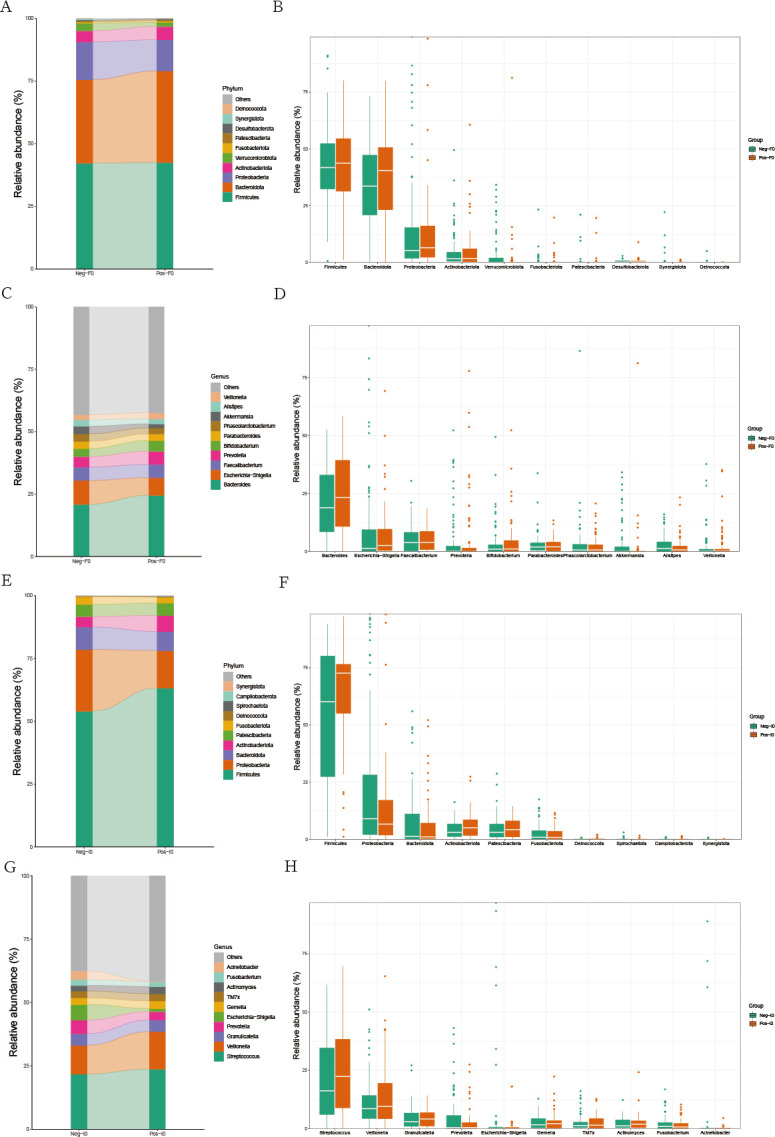
Bar plot and box plot of the top 10 most abundant species at different levels of the colon microbiota and small intestinal microbiota at different levels in two groups of patients at baseline. (**A** and **B**) Phylum level in the colon microbiota. (**C** and **D**) Genus level in the colon microbiota. (**E** and **F**) Phylum level in the small intestinal microbiota. (**G** and **H**) Genus level in the small intestinal microbiota. “Neg” represents the non-SIBO group, “Pos” represents the SIBO group. “F” denotes feces, “I” denotes small intestinal fluid. “0” represents the baseline state.

To delve deeper into the determinants influencing the effectiveness of FMT, we conducted a comparative analysis of the baseline microbial profiles in the colonic and small intestinal microbiota across two patient cohorts. Our findings revealed minimal disparities in the colonic microbial compositions between these groups (Fig. 4A through D). However, there was a larger difference in the small intestinal microbiota. At the phylum level, the top 4 abundant phyla were *Firmicutes*, *Proteobacteria*, *Bacteroidota*, and *Actinobacteriota*. Compared with the non-SIBO group, the SIBO group had a higher abundance of *Firmicutes* and a lower abundance of *Proteobacteria* (Fig. 4E and F). At the genus level, the abundance of *Veillonella* in the SIBO group (13.90%) was higher than that in the non-SIBO group (11.30%), while the abundances of *Escherichia-Shigella* and *Acinetobacter* were lower than those in the non-SIBO group. The abundance of *Escherichia-Shigella* was 6.10% in the non-SIBO group and 1.17% in the SIBO group, and the abundance of *Acinetobacter* was 3.41% in the non-SIBO group and 0.26% in the SIBO group (Fig. 4G and H).

**Fig 5:**
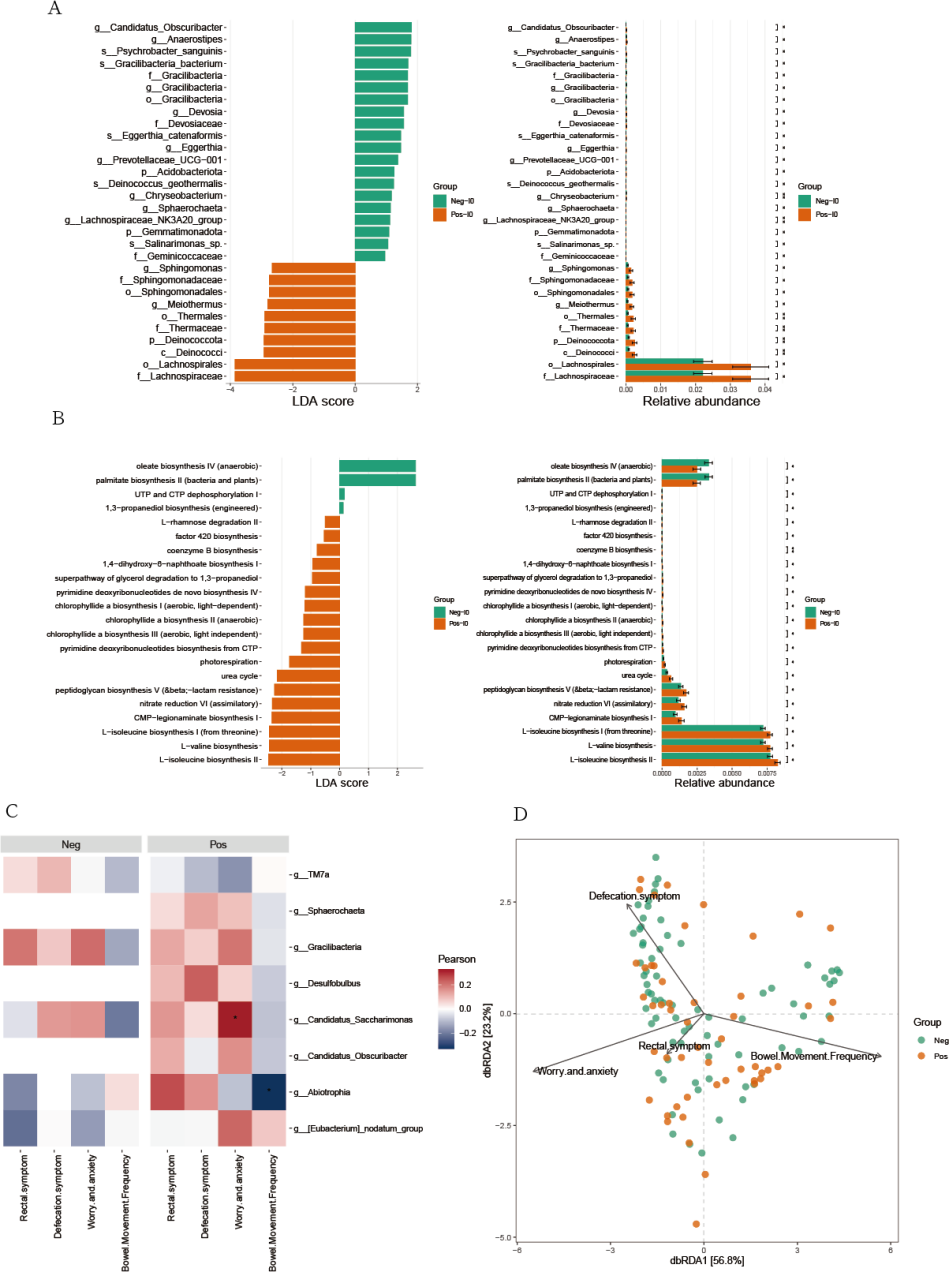
LEfSe analysis and correlation between differentially abundant species and clinical features in the small intestinal microbiota. (**A**) Gut microbiota differences between the small intestinal microbiota of two groups were identified with a LEfSe analysis with an LDA score threshold >2.0. (**B**) KEGG level 2 annotation for the differently abundant function pathway in small intestinal microbiota of the two groups. The heat (C) and branching maps (D) of correlations between clinical characteristics and differentially abundant ASVs identified by LEfSe analysis. “Neg” represents the non-SIBO group, “Pos” represents the SIBO group. “I” denotes small intestinal fluid. “0” represents the baseline state.

To further explore how the baseline state of the small intestinal fluid influences the efficacy of FMT, we performed LEfSe analysis on the baseline samples from the two groups of patients. It was found that *Candidatus_Obscuribacter*, *Anaerostipes*, and *Gracilibacteria* were enriched in the non-SIBO group, while *Sphingomonas* and *Meiothermus* were enriched in the SIBO group (Fig. 5A). Additionally, functional predictions were made on the baseline small intestinal fluid, revealing stronger biosynthesis capabilities for isoleucine and valine in the SIBO group (Fig. 5B). Previous studies have shown that isoleucine and valine contribute to increased muscle protein synthesis (30), which in turn can enhance intestinal motility to some extent, explaining why FMT is more effective in constipated patients with SIBO. We further analyzed the correlation between the differentially enriched species in the small intestinal fluid and clinical characteristics. The results indicated that *Candidatus_Saccharimonas* in the SIBO group was significantly correlated with worry and anxiety (*P* < 0.05), and *Abiotrophia* in the SIBO group was significantly correlated with bowel movement frequency (*P* < 0.05) (Fig. 5C and D).

## DISCUSSION

SIBO is a gastrointestinal disorder characterized by an increased number of bacteria and/or abnormal types in the small intestine. It is considered a secondary disease due to changes in intestinal anatomical structure, reduced intestinal motility, and gastrointestinal dysfunction (31). These bacteria mainly include Gram-negative anaerobes and aerobes, which, under certain conditions, can overgrow, leading to symptoms such as diarrhea, weight loss, and malnutrition (32). In this study, there was no difference in the AEs experienced by the two groups of constipated patients during the FMT process, reflecting the safety of FMT treatment for SIBO patients. Moreover, a randomized controlled trial also confirmed the safety of FMT for patients with SIBO (33).

In this study, constipation-related symptoms, including abdominal, rectal, and bowel symptoms, were assessed using specific scales before and after FMT in patients. It was observed that patients with constipation and SIBO exhibited markedly better clinical outcomes compared to constipation patients without SIBO. To investigate potential mechanisms, 16S rRNA sequencing was conducted on fecal and small intestinal fluid samples from both patient groups. The results showed minimal changes in the colonic microbiota post-FMT, whereas significant differences were noted in the small intestinal microbiota. At the genus level, both groups demonstrated a significant reduction in the abundance of *Streptococcus* and *Veillonella* after FMT, but an increase in the abundance of *Escherichia-Shigella*. Studies have reported that FMT can ameliorate the disruption of gut microbial diversity caused by antibiotics, restore the abundance of various anaerobes, and reduce the abundance of genera such as *Enterococcus*, *Streptococcus*, and *Veillonella* (34). A double-blind trial of FMT for ulcerative colitis revealed a negative correlation between the abundance of *Streptococcus* in donor stool and patient remission rates (35). An analysis of microbiota data from 106 patients with primary sclerosing cholangitis and IBD, compared to healthy individuals, suggested a link between *Veillonella* and IBD (36). *Veillonella* can utilize nitrate, a hallmark metabolic byproduct of inflammation, for anaerobic respiration as a terminal electron acceptor, relying on the narGHJI operon for nitrate respiration, which promotes its growth in organic acids and allows it to utilize amino acids and peptides as carbon sources (37). An increase in *Escherichia-Shigella* abundance was found in stool samples from patients with antibiotic-associated diarrhea and chronic diarrhea (38). These bacteria can invade intestinal cells from the lumen side, compromising gut barrier integrity and rapidly colonizing crypt-like invaginations, enhancing their adhesion (39). Additionally, research has found that besides the core microbiome, *Shigella* is commonly present in the duodenum and jejunum (40). The same microbial population can play different roles in different physiological parts of the intestine. As the gut environment changes from the duodenum to the colon, this promotes the function-specific bacteria to act in specific locations (40).

To further investigate the impact of SIBO on the efficacy of FMT in treating chronic constipation, an exploration of the baseline state of the colonic and small intestinal microbiota in two patient groups was conducted. It was discovered that there were no significant differences in the fecal microbiota between the two groups at baseline; however, clear distinctions were present in the microbial composition of the small intestinal fluid. Studies analyzing 316 clinical and metagenomic data sets of FMT found that the pre-FMT microbial structure and diversity, as well as the donor-recipient microbial complementarity, determined the restoration capacity and effectiveness of FMT (41). The recipient’s original gut microbiota may affect the colonization of introduced donor microbes through energy competition, ecological niche competition, and immune interactions (42). Patients with constipation and SIBO had a significantly higher abundance of *Streptococcus* and *Veillonella* in their small intestinal fluid compared to the non-SIBO constipation group, with a notably lower abundance of *Escherichia-Shigella*, indicating that the baseline state of the small intestinal microbiota may affect the efficacy of FMT. Studies have linked *Escherichia-Shigella* with carbohydrate-related metabolism and various lipid-related metabolites, and *Veillonella* can metabolize lactate into propionate (43, 44). FMT has been shown to alter the relative abundance of obligate anaerobes in the gut and also to modify carbohydrate metabolic pathways, thereby reducing the incidence of necrotizing enterocolitis (45). Therefore, we speculate that the different therapeutic outcomes observed in the two constipated patient groups may be related to the baseline state of the small intestinal fluid microbiota, particularly in relation to carbohydrate metabolism.

The small intestine is a critical site for nutrient absorption in the human body, yet its microbiota remains an area in urgent need of exploration. Existing studies have linked the small intestinal microbiota to functional gastrointestinal diseases, immune system disorders, and metabolism-related diseases (40). One study revealed that compared to healthy controls, patients with Crohn’s disease exhibited a significant increase in alpha-diversity of the duodenal mucosal microbiota, along with notable alterations in the abundance of 24 bacterial genera including *Streptococcus* in the duodenum and ileum (46). Another study analyzed the duodenal microbiome of 36 children with environmental enteric dysfunction and identified 14 core bacterial taxa not typically considered enteric pathogens. These bacteria, including certain *Veillonella* species, *Streptococcus*, and *Rothia mucilaginosa*, showed an inverse correlation with the children’s growth and a positive correlation with duodenal proteins involved in immune and inflammatory responses, such as Lipocalin-2 (LCN2), and their abundance was distinct from that found in healthy children’s stool (47). Increasing evidence suggests a close relationship between the small intestinal microbiota and human health, and the findings of this research also indicate that the state of the small intestinal microbiota can affect the therapeutic effect of FMT. Research has shown that whole-gut microbiota transplantation is more precise than FMT, with animal experiments revealing that specific segments of the donor’s microbiota and their functions tend to colonize and exert their effects in the corresponding segments of the recipient’s gut. For instance, *Proteobacteria*, *Lactobacillaceae*, and *Cyanobacteria* from the donor’s small intestine are likely to colonize in the recipient’s small intestine, while anaerobes degrading carbohydrates from the donor’s colon are prone to settle in the recipient’s colon (48). However, due to the limitations in sample collection, further exploration is required in the study of the small intestinal microbiota.

This study has certain limitations. Firstly, the examination of patients’ fecal and small intestine fluid samples was conducted using the 16S rRNA sequencing method, and our current functionality is based on 16S rRNA sequencing with KEGG prediction. As a next step, we plan to conduct metagenomic and metabolomics sequencing on the samples. Additionally, in this study, during the process of collecting samples, we used a negative pressure device to draw out the small intestinal fluid through a nasojejunal tube. This method may have caused a certain degree of contamination to the small intestinal fluid samples. Subsequently, we will improve the method for collecting small intestinal fluid samples.

## Data Availability

The 16S rRNA sequencing data of all participants in the study has been uploaded to the National Center for Biotechnology Information platform, with the accession number PRJNA1099433

## References

[1] Pimentel M, Saad RJ, Long MD, Rao SSC. 2020. ACG clinical guideline: small intestinal bacterial overgrowth. Am J Gastroenterol 115:165–178. doi:10.14309/ajg.0000000000000501 32023228

[2] Ghoshal UC, Ghoshal U. 2017. Small intestinal bacterial overgrowth and other intestinal disorders. Gastroenterol Clin North Am 46:103–120. doi:10.1016/j.gtc.2016.09.008 28164845

[3] Polkowska-Pruszyńska B, Gerkowicz A, Szczepanik-Kułak P, Krasowska D. 2019. Small intestinal bacterial overgrowth in systemic sclerosis: a review of the literature. Arch Dermatol Res 311:1–8. doi:10.1007/s00403-018-1874-0 30382339 PMC6326989

[4] Rezaie A, Buresi M, Lembo A, Lin H, McCallum R, Rao S, Schmulson M, Valdovinos M, Zakko S, Pimentel M. 2017. Hydrogen and methane-based breath testing in gastrointestinal disorders: the North American consensus. Am J Gastroenterol 112:775–784. doi:10.1038/ajg.2017.46 28323273 PMC5418558

[5] Levitt MD, Bond JH. 1970. Volume, composition, and source of intestinal gas. Gastroenterology 59:921–929. doi:10.1016/S0016-5085(19)33654-6 5486278

[6] Gasbarrini A, Corazza GR, Gasbarrini G, Montalto M, Di Stefano M, Basilisco G, Parodi A, Usai-Satta P, Vernia P, Anania C, et al.. 2009. Methodology and indications of H2-breath testing in gastrointestinal diseases: the Rome consensus conference. Aliment Pharmacol Ther 29 Suppl 1:1–49. doi:10.1111/j.1365-2036.2009.03951.x 19344474

[7] Bharucha AE, Pemberton JH, Locke GR. 2013. American gastroenterological association technical review on constipation. Gastroenterology 144:218–238. doi:10.1053/j.gastro.2012.10.028 23261065 PMC3531555

[8] Tamura A, Tomita T, Oshima T, Toyoshima F, Yamasaki T, Okugawa T, Kondo T, Kono T, Tozawa K, Ikehara H, Ohda Y, Fukui H, Watari J, Miwa H. 2016. Prevalence and self-recognition of chronic constipation: results of an internet survey. J Neurogastroenterol Motil 22:677–685. doi:10.5056/jnm15187 27426278 PMC5056578

[9] Lembo A, Camilleri M. 2003. Chronic constipation. N Engl J Med 349:1360–1368. doi:10.1056/NEJMra020995 14523145

[10] Parthasarathy G, Chen J, Chen X, Chia N, O’Connor HM, Wolf PG, Gaskins HR, Bharucha AE. 2016. Relationship between microbiota of the colonic mucosa vs feces and symptoms, colonic transit, and methane production in female patients with chronic constipation. Gastroenterology 150:367–379. doi:10.1053/j.gastro.2015.10.005 26460205 PMC4727996

[11] Khalif I, Quigley E, Konovitch E, Maximova I. 2005. Alterations in the colonic flora and intestinal permeability and evidence of immune activation in chronic constipation. Dig Liver Dis 37:838–849. doi:10.1016/j.dld.2005.06.008 16169298

[12] Zhuang M, Shang W, Ma Q, Strappe P, Zhou Z. 2019. Abundance of probiotics and butyrate‐production microbiome manages constipation via short‐chain fatty acids production and hormones secretion. Mol Nutr Food Res 63:e1801187. doi:10.1002/mnfr.201801187 31556210

[13] Ge X, Zhao W, Ding C, Tian H, Xu L, Wang H, Ni L, Jiang J, Gong J, Zhu W, Zhu M, Li N. 2017. Potential role of fecal microbiota from patients with slow transit constipation in the regulation of gastrointestinal motility. Sci Rep 7:441. doi:10.1038/s41598-017-00612-y 28348415 PMC5428802

[14] Porcari S, Benech N, Valles-Colomer M, Segata N, Gasbarrini A, Cammarota G, Sokol H, Ianiro G. 2023. Key determinants of success in fecal microbiota transplantation: from microbiome to clinic. Cell Host Microbe 31:712–733. doi:10.1016/j.chom.2023.03.020 37167953

[15] Halfvarson J, Brislawn CJ, Lamendella R, Vázquez-Baeza Y, Walters WA, Bramer LM, D’Amato M, Bonfiglio F, McDonald D, Gonzalez A, McClure EE, Dunklebarger MF, Knight R, Jansson JK. 2017. Dynamics of the human gut microbiome in inflammatory bowel disease. Nat Microbiol 2:17004. doi:10.1038/nmicrobiol.2017.4 28191884 PMC5319707

[16] de Groot PF, Frissen MN, de Clercq NC, Nieuwdorp M. 2017. Fecal microbiota transplantation in metabolic syndrome: history, present and future. Gut Microbes 8:253–267. doi:10.1080/19490976.2017.1293224 28609252 PMC5479392

[17] Gupta A, Cifu AS, Khanna S. 2018. Diagnosis and treatment of Clostridium difficile infection. JAMA 320:1031–1032. doi:10.1001/jama.2018.12194 30178042

[18] Hinton DE, Lantz RC, Hampton JA, McCuskey PR, McCuskey RS. 1987. Normal versus abnormal structure: considerations in morphologic responses of teleosts to pollutants. Environ Health Perspect 71:139–146. doi:10.1289/ehp.8771139 3297656 PMC1474344

[19] Ge X, Tian H, Ding C, Gu L, Wei Y, Gong J, Zhu W, Li N, Li J. 2016. Fecal microbiota transplantation in combination with soluble dietary fiber for treatment of slow transit constipation: a pilot study. Arch Med Res 47:236–242. doi:10.1016/j.arcmed.2016.06.005 27387020

[20] El-Salhy M, Winkel R, Casen C, Hausken T, Gilja OH, Hatlebakk JG. 2022. Efficacy of fecal microbiota transplantation for patients with irritable bowel syndrome at 3 years after transplantation. Gastroenterology 163:982–994. doi:10.1053/j.gastro.2022.06.020 35709830

[21] Ding C, Fan W, Gu L, Tian H, Ge X, Gong J, Nie Y, Li N. 2018. Outcomes and prognostic factors of fecal microbiota transplantation in patients with slow transit constipation: results from a prospective study with long-term follow-up. Gastroenterol Rep (Oxf) 6:101–107. doi:10.1093/gastro/gox036 29780597 PMC5952918

[22] Yamada E, Tsunoda S, Mimura M, Akizuki M, Miyazawa Y, Yamazaki T, Nagano Y, Murakami R, Kitahara T, Wakasugi J, Ozawa Y, Komatsu T, Inamori M, Nagai K, Nakajima A. 2021. Positioning of bristol stool form scale type 3 in constipation treatment satisfaction: a multicenter study in Japan. J Gastroenterol Hepatol 36:2125–2130. doi:10.1111/jgh.15428 33538361

[23] Agachan F, Chen T, Pfeifer J, Reissman P, Wexner SD. 1996. A constipation scoring system to simplify evaluation and management of constipated patients. Dis Colon Rectum 39:681–685. doi:10.1007/BF02056950 8646957

[24] Altomare DF, Picciariello A, Di Ciaula A, Rinaldi M, De Fazio M, Portincasa P. 2021. Effects of temporary sacral nerve stimulation on gastrointestinal motility and function in patients with chronic refractory slow-transit constipation. Tech Coloproctol 25:291–297. doi:10.1007/s10151-020-02367-7 33185809 PMC7932968

[25] Bolyen E, Rideout JR, Dillon MR, Bokulich NA, Abnet CC, Al-Ghalith GA, Alexander H, Alm EJ, Arumugam M, Asnicar F, et al.. 2019. Reproducible, interactive, scalable and extensible microbiome data science using QIIME 2. Nat Biotechnol 37:852–857. doi:10.1038/s41587-019-0209-9 31341288 PMC7015180

[26] Callahan BJ, McMurdie PJ, Rosen MJ, Han AW, Johnson AJA, Holmes SP. 2016. DADA2: High-resolution sample inference from Illumina amplicon data. Nat Methods 13:581–583. doi:10.1038/nmeth.3869 27214047 PMC4927377

[27] Liu C, Cui Y, Li X, Yao M. 2021. Microeco: an R package for data mining in microbial community ecology. FEMS Microbiol Ecol 97:fiaa255. doi:10.1093/femsec/fiaa255 33332530

[28] Segata N, Izard J, Waldron L, Gevers D, Miropolsky L, Garrett WS, Huttenhower C. 2011. Metagenomic biomarker discovery and explanation. Genome Biol 12:R60. doi:10.1186/gb-2011-12-6-r60 21702898 PMC3218848

[29] Kanehisa M, Goto S, Sato Y, Furumichi M, Tanabe M. 2012. KEGG for integration and interpretation of large-scale molecular data sets. Nucleic Acids Res 40:D109–14. doi:10.1093/nar/gkr988 22080510 PMC3245020

[30] White PJ, Newgard CB. 2019. Branched-chain amino acids in disease. Science 363:582–583. doi:10.1126/science.aav0558 30733403 PMC9940269

[31] Skrzydło-Radomańska B, Cukrowska B. 2022. How to recognize and treat small intestinal bacterial overgrowth? J Clin Med 11:6017. doi:10.3390/jcm11206017 36294338 PMC9604644

[32] Ghoshal UC, Sachdeva S, Ghoshal U, Misra A, Puri AS, Pratap N, Shah A, Rahman MM, Gwee KA, Tan VPY, Ahmed T, Lee YY, Ramakrishna BS, Talukdar R, Rana SV, Sinha SK, Chen M, Kim N, Holtmann G. 2022. Asian-Pacific consensus on small intestinal bacterial overgrowth in gastrointestinal disorders: an initiative of the Indian neurogastroenterology and motility association. Indian J Gastroenterol 41:483–507. doi:10.1007/s12664-022-01292-x 36214973 PMC9549446

[33] Xu F, Li N, Wang C, Xing H, Chen D, Wei Y. 2021. Clinical efficacy of fecal microbiota transplantation for patients with small intestinal bacterial overgrowth: a randomized, placebo-controlled clinic study. BMC Gastroenterol 21:54. doi:10.1186/s12876-021-01630-x 33549047 PMC7866462

[34] Rashidi A, Ebadi M, Rehman TU, Elhusseini H, Kazadi D, Halaweish H, Khan MH, Hoeschen A, Cao Q, Luo X, Kabage AJ, Lopez S, Holtan SG, Weisdorf DJ, Khoruts A, Staley C. 2023. Randomized double-blind phase II trial of fecal microbiota transplantation versus placebo in allogeneic hematopoietic cell transplantation and AML. J Clin Oncol 41:5306–5319. doi:10.1200/JCO.22.02366 37235836 PMC10691796

[35] Paramsothy S, Nielsen S, Kamm MA, Deshpande NP, Faith JJ, Clemente JC, Paramsothy R, Walsh AJ, van den Bogaerde J, Samuel D, Leong RWL, Connor S, Ng W, Lin E, Borody TJ, Wilkins MR, Colombel J-F, Mitchell HM, Kaakoush NO. 2019. Specific bacteria and metabolites associated with response to fecal microbiota transplantation in patients with ulcerative colitis. Gastroenterology 156:1440–1454. doi:10.1053/j.gastro.2018.12.001 30529583

[36] Vieira-Silva S, Sabino J, Valles-Colomer M, Falony G, Kathagen G, Caenepeel C, Cleynen I, van der Merwe S, Vermeire S, Raes J. 2019. Quantitative microbiome profiling disentangles inflammation- and bile duct obstruction-associated microbiota alterations across PSC/IBD diagnoses. Nat Microbiol 4:1826–1831. doi:10.1038/s41564-019-0483-9 31209308

[37] Rojas-Tapias DF, Brown EM, Temple ER, Onyekaba MA, Mohamed AMT, Duncan K, Schirmer M, Walker RL, Mayassi T, Pierce KA, Ávila-Pacheco J, Clish CB, Vlamakis H, Xavier RJ. 2022. Inflammation-associated nitrate facilitates ectopic colonization of oral bacterium Veillonella parvula in the intestine. Nat Microbiol 7:1673–1685. doi:10.1038/s41564-022-01224-7 36138166 PMC9728153

[38] DuPont HL. 2016. Persistent diarrhea: a clinical review. JAMA 315:2712–2723. doi:10.1001/jama.2016.7833 27357241

[39] Grassart A, Malardé V, Gobaa S, Sartori-Rupp A, Kerns J, Karalis K, Marteyn B, Sansonetti P, Sauvonnet N. 2019. Bioengineered human organ-on-chip reveals intestinal microenvironment and mechanical forces impacting Shigella infection. Cell Host Microbe 26:435–444. doi:10.1016/j.chom.2019.08.007 31492657

[40] Yersin S, Vonaesch P. 2024. Small intestinal microbiota: from taxonomic composition to metabolism. Trends Microbiol. doi:10.1016/j.tim.2024.02.013 38503579

[41] Schmidt TSB, Li SS, Maistrenko OM, Akanni W, Coelho LP, Dolai S, Fullam A, Glazek AM, Hercog R, Herrema H, Jung F, Kandels S, Orakov A, Thielemann R, von Stetten M, Van Rossum T, Benes V, Borody TJ, de Vos WM, Ponsioen CY, Nieuwdorp M, Bork P. 2022. Drivers and determinants of strain dynamics following fecal microbiota transplantation. N Med 28:1902–1912. doi:10.1038/s41591-022-01913-0 PMC9499871 36109636

[42] Danne C, Rolhion N, Sokol H. 2021. Recipient factors in faecal microbiota transplantation: one stool does not fit all. Nat Rev Gastroenterol Hepatol 18:503–513. doi:10.1038/s41575-021-00441-5 33907321

[43] Wu Z, Zhang Q, Yang J, Zhang J, Fu J, Dang C, Liu M, Wang S, Lin Y, Hao J, Weng M, Xie D, Li A. 2022. Significant alterations of intestinal symbiotic microbiota induced by intraperitoneal vaccination mediate changes in intestinal metabolism of NEW genetically improved farmed Tilapia (NEW GIFT, Oreochromis niloticus). Microbiome 10:221. doi:10.1186/s40168-022-01409-6 36510260 PMC9742657

[44] Scheiman J, Luber JM, Chavkin TA, MacDonald T, Tung A, Pham L-D, Wibowo MC, Wurth RC, Punthambaker S, Tierney BT, Yang Z, Hattab MW, Avila-Pacheco J, Clish CB, Lessard S, Church GM, Kostic AD. 2019. Meta-omics analysis of elite athletes identifies a performance-enhancing microbe that functions via lactate metabolism. N Med 25:1104–1109. doi:10.1038/s41591-019-0485-4 PMC7368972 31235964

[45] Brunse A, Martin L, Rasmussen TS, Christensen L, Skovsted Cilieborg M, Wiese M, Khakimov B, Pieper R, Nielsen DS, Sangild PT, Thymann T. 2019. Effect of fecal microbiota transplantation route of administration on gut colonization and host response in preterm pigs. ISME J 13:720–733. doi:10.1038/s41396-018-0301-z 30367124 PMC6461782

[46] Wauters L, Tito RY, Ceulemans M, Outtier A, Rymenans L, Verspecht C, Sabino J, Ferrante M, Vermeire S, Vanuytsel T, Raes J. 2023. Microbiome variance of the small bowel in Crohn’s disease. Gut 72:1626–1628. doi:10.1136/gutjnl-2022-327313 37414433

[47] Chen RY, Kung VL, Das S, Hossain MS, Hibberd MC, Guruge J, Mahfuz M, Begum S, Rahman MM, Fahim SM, Gazi MA, Haque R, Sarker SA, Mazumder RN, Di Luccia B, Ahsan K, Kennedy E, Santiago-Borges J, Rodionov DA, Leyn SA, Osterman AL, Barratt MJ, Ahmed T, Gordon JI. 2020. Duodenal microbiota in stunted undernourished children with enteropathy. N Engl J Med 383:321–333. doi:10.1056/NEJMoa1916004 32706533 PMC7289524

[48] Li N, Zuo B, Huang S, Zeng B, Han D, Li T, Liu T, Wu Z, Wei H, Zhao J, Wang J. 2020. Spatial heterogeneity of bacterial colonization across different gut segments following inter-species microbiota transplantation. Microbiome 8:161. doi:10.1186/s40168-020-00917-7 33208178 PMC7677849

